# The association between 12-hour shifts and nurses-in-charge's perceptions of missed care and staffing adequacy: a retrospective cross-sectional observational study

**DOI:** 10.1016/j.ijnurstu.2020.103721

**Published:** 2020-12

**Authors:** Christina Saville, Chiara Dall'Ora, Peter Griffiths

**Affiliations:** School of Health Sciences, The University of Southampton, UK

**Keywords:** 12‐hr shifts, health resources, nurses, personnel staffing and scheduling, quality of healthcare, shift work schedule

## Abstract

**Background:**

Due to worldwide nursing shortages and difficulty retaining staff, long shifts for nursing staff (both registered nurses and nursing assistants) working in hospitals have been adopted widely. Because long shifts reduce the daily number of shifts from three to two, many assume that long shifts improve productivity by removing one handover and staff overlap. However, it is unclear whether staffing levels are more likely to be perceived as adequate when more long shifts are used.

**Objectives:**

To investigate the association between the proportion of long (≥12‐hour) shifts worked on a ward and nurses-in-charge's perceptions that the staffing level was sufficient to meet patient need.

**Methods:**

A retrospective cross-sectional study using routinely collected data (patient administrative data and rosters) linked to nurses-in-charge's reports from 81 wards within four English hospitals across 1 year (2017). Hierarchical logistic regression models were used to determine associations between the proportion of long shifts and nurses-in-charge's reports of having enough staff for quality or leaving necessary nursing care undone, after controlling for the staffing level relative to demand (shortfall). We tested for interactions between staffing shortfall and the proportion of long shifts.

**Results:**

The sample comprised 19648 ward days. On average across wards, 72% of shifts were long. With mixed short and long shifts, the odds of nurses-in-charge reporting that there were enough staff for quality were 14-17% lower than when all shifts were long. For example, the odds of reporting enough staff for quality with between 60-80% long shifts was 15% lower (95% confidence interval 2% to 27%) than with all long shifts. Associations with nursing care left undone were consistent with this pattern. Although including interactions between staffing shortfalls and the proportion of long shifts did not improve model fit, the effect of long shifts did appear to differ according to shortfall, with lower proportions of long shifts associated with benefits when staffing levels were high relative to current norms.

**Conclusions:**

Rather than a clear distinction between wards using short and long shifts, we found that a mixed pattern operated on most days and wards, with no wards using all short shifts. We found that when wards use exclusively long shifts rather than a mixture, nurses-in-charge are more likely to judge that they have enough staff. However, the adverse effects of mixed shifts on perceptions of staffing adequacy may be reduced or eliminated by higher staffing levels.

ISRCTN 12307968.

**Tweetable abstract**

12-hour shifts in nursing: a mix of short and long shifts may be worse than all long shifts.

**What is already known about the topic?**

• Working long shifts is associated with nurse-reported lower quality of care and more care left undone, as well as staff fatigue, sickness absence, burnout and intention to leave.
• Some staff prefer working long shifts.
• Despite claims of increased efficiency, a previous study found that compared to all short shifts, long shifts did not reduce resource use, while mixtures of short and long shifts were more resource-intensive.

**What this paper adds**

• This analysis provides evidence for a non-linear relationship between the proportion of long shifts and the perceived adequacy of nurse staffing.
• We found evidence that nurses-in-charge were less likely to perceive staffing as adequate when a mixture of short and long shifts were used compared to all long shifts.
• The effect of long shifts may depend on baseline staffing levels.

## 1 Introduction

Worldwide there are too few qualified nurses and many hospitals are struggling to retain them (Mitchell, 2019, World Health Organization, 2013). Therefore it is important that hospitals make the best use of the nurses they have to provide quality patient care. An unresolved global problem is how best to organise shifts for nursing staff (both registered nurses and nursing assistants) working on hospital inpatient wards. A particular issue is whether long (≥12-hour) shifts are a more efficient use of staff than shorter shifts. Utilising long instead of short shifts has for some time been advocated as a way of reducing handover time and overlaps (Ganong et al., 1976). Long shifts can be used to reduce the daily number of shifts from three to two, requiring one less handover and reducing staff overlaps between shifts. Effectively, 12-hour shifts are presented as improving productivity by removing unproductive time, which would therefore mean that adequate staffing could be maintained with fewer total hours of care.

Despite claims of benefits, existing evidence does not support the premise that long shifts are more productive than short shifts. Working long shifts is associated with a higher risk of sickness absence, suggesting that assigning staff to long shifts is not an efficient use of the workforce (Dall'Ora et al., 2019a). A study at one English hospital found no cost savings and the same resource use when more than 75% of worked shifts were long as when no worked shifts were long, while mixed shift patterns (more than 0 and less than 75% long shifts) led to higher costs and resource use (Griffiths et al., 2019). Such mixed shift patterns potentially undermine benefits of reducing handovers by increasing the potential number of handovers required and / or increasing the risk that important information is not passed on. There is some evidence that long shifts may remove the chance for educational and communication activities that occur during overlaps, Dall'Ora et al., 2019b both pivotal to enable nursing staff to provide safe and effective care. Furthermore, large multi-site studies have found that registered nurses working long shifts is associated with more errors (Rogers et al., 2004), more care left undone (Ball et al., 2017, Griffiths et al., 2014), lower self-reported quality of care (Ball et al., 2017, Griffiths et al., 2014) and more occurrences of self-reported poor or failing patient safety (Griffiths et al., 2014). The proportion of long nursing assistant shifts is associated with delayed vital signs observations Dall'Ora et al., 2019c.

Different shift patterns are in use across different countries and hospitals (Garde et al., 2019, Griffiths et al., 2014) and even across wards in the same hospital (Ball et al., 2017). Wards may introduce 12-hour shifts because of the assumption that they improve continuity of care and reduce costs (NHS evidence, 2010). Shift patterns have implications for staff wellbeing; working long shifts is associated with fatigue, (Thompson, 2019) sickness absence, (Dall'Ora et al., 2019a) burnout (Dall'Ora et al., 2015) and intention to leave, (Dall'Ora et al., 2015) but some staff prefer working long shifts (Baillie and Thomas, 2019, Ose et al., 2019). In the UK, nurses’ satisfaction with the choice of length of shifts is declining, (Marangozov et al., 2017) which is particularly concerning given the afore-mentioned problems with staff retention. To make jobs more attractive, managers may offer staff more shift length options.

In this study we investigate whether the use of long nursing shifts is associated with changes in the perceptions of nurses-in-charge that nurse staffing is adequate (i.e. that there are enough registered nurses and nursing assistants to do the work). We used two measures of staffing adequacy: whether there were enough staff to provide quality care, and whether necessary nursing care was left undone (missed) because there were too few nursing staff. Specifically, we test the hypothesis that long shifts increase the likelihood that any given staffing level is associated with reports of adequate staffing (i.e. enough staff for quality and no missed care). We also test whether the size of staffing shortfalls affects the relationship between the use of long shifts and staffing adequacy.

## 2 Methods

### 2.1 Setting and inclusion criteria

This retrospective cross-sectional observational study was undertaken in the National Health Service (NHS), the publicly funded healthcare system in England, which is free at the point of delivery and provides the vast majority of acute health care, both emergency and elective. Funding is allocated to providers, primarily based on planned activity levels using a diagnosis-related-group pricing system. The study sample consists of 81 wards from two general hospitals, one university teaching hospital and one specialist cancer hospital (spread across two sites) in London, South East and South West England. These serve different types of patient populations including deprived inner-city populations, rural regions and specialist national referrals. The hospitals use a mixture of long and short nursing shifts, which is typical of hospitals in England where there has been a move from 8-hour to more use of 12-hour day shifts over the last 40 years (Griffiths et al., 2019).

We included general medical and surgical inpatient wards that care for patients 24 hours a day, seven days a week. We measured staffing requirements using the Safer Nursing Care Tool, so excluded wards that are out of scope of this tool (The Shelford group, 2014) (e.g. paediatrics, intensive care, maternity, neonatal and palliative care), and any others that have highly abnormal staffing requirements (e.g. bone marrow transplant and isolation units). This sample of wards represents 74% of the beds across the four hospitals. We previously used this same sample to investigate whether there was an association between staffing deficits as measured by the Safer Nursing Care Tool and nurses-in-charge's perceptions of staffing adequacy, (Griffiths et al., 2020a, Griffiths et al., 2020b) and for developing a simulation to assess the costs and consequences of different staffing levels (Griffiths et al., 2020a, Saville et al., 2020).

### 2.2 Data sources and measures

Our data covered a period of one year (2017). We linked together data from multiple sources at the ward day level and checked the number of records before and after linkage. We identified the lengths of shifts and hours worked from routine roster data. We used patient administration data for the patient admissions and discharges. Each hospital supplied additional information about the wards (the main speciality and layout including the number of beds and single rooms).

At least twice per day, the nurse-in-charge recorded the numbers of patients in each Safer Nursing Care Tool acuity/dependency category and their professional judgements about the adequacy of overall nurse staffing (both registered nurses and nursing assistants; see details below). At each hospital, potential nurses-in-charge on participating wards were trained in using the Safer Nursing Care Tool and completion of the staffing adequacy questions. Laminated sheets providing supporting information and brief guidance were kept near the ward computers where data were entered. As part of pilot work in one Trust, we assessed inter-rater reliability of the Safer Nursing Care Tool between an expert and 15 trained nurses-in-charge working across 14 wards. In total 847 patients were rated independently by two raters across 81 shifts. At the patient level, there was 89% agreement on ratings (kappa 0.71 – moderate agreement). At the shift level, there was a mean difference of +/-1.9% in the estimated staff whole-time-equivalents.

### 2.3 Study variables

For each ward and day, we calculated the proportion of hours between 7am-7pm that were from long (≥12‐hour) shifts. For this, we considered shifts worked by any nursing staff, both registered nurses and nursing assistants. We considered the proportion of long shifts as a categorical variable, since there is some evidence from existing literature that effects may not be linear, but that mixed shift patterns may be the most resource-intensive and expensive (Griffiths et al., 2019).

We estimated the overall required staffing (both registered nurses and nursing assistants) using the Safer Nursing Care Tool, which is widely used in England to set nurse staffing levels. This tool works by categorising patients into levels (0, 1a, 1b, 2 or 3) according to their acuity and dependency on nursing care. Each level has an associated “multiplier”, which represents the nursing staff employed to ensure adequate care for a patient in that level. We used the most recent version of the Safer Nursing Care Tool, including the multipliers that were in use at the start of the study (The Shelford group, 2014).

Since the Safer Nursing Care Tool estimate of the staff required is measured in whole-time-equivalents, we converted this into the hours of staff time required each daytime from 7am – 7pm in the following way. From the nurses-in-charge's reports of numbers of patients per acuity/dependency category, we calculated the weighted average multiplier per ward for each morning and afternoon. We used the record at the beginning of the period or the end if missing. In case any patients were omitted from the reports, we multiplied this by the total patient count derived from patient administration systems at 7am/1pm. There was close correspondence between the overall numbers of patients reported using the SNCT and the patient counts derived from patient administration systems, with under-reporting of one patient on average (Griffiths et al., 2020a). Since the multipliers are designed to provide an estimate of the required number of staff to employ, we converted this to the implied hours for the daytime from 7am – 7pm. For this, we used a 37.5 hour working week for 1 whole-time-equivalent, removing the 22% ‘uplift’, which is added in the tool to account for study and sick leave, and weighted by each ward's average distribution of staff over day (7am-7pm) versus night (7pm-7am). The Safer Nursing Care Tool does not directly account for patients identified as requiring 1-to-1 supervision, often referred to as ‘specialing’ (Wood et al., 2018), so we identified the number of such patients from records and added the required hours to our estimated staffing requirement. We used the average observed skill mix on each ward as a proxy for the planned skill mix of registered nurses and nursing assistants.

We calculated worked hours per patient day for each ward for each day by identifying hours worked (i.e. excluding breaks) by registered nurses and nursing assistants each day (from 7am to 7pm) from the electronic roster and dividing these by the number of patient days (patient hours / 24). We calculated the shortfall per patient day by subtracting the required hours from the hours deployed on that day. If more staff than the estimated requirement were deployed, the shortfall was negative.

We also calculated daily patient turnover per staff member (the numbers of patients entering and leaving wards divided by the total staff hours from 7am-7pm).

Outcomes were nurses-in-charge's reports of the adequacy of overall nurse staffing (both registered nurses and nursing assistants). These were based on the widely used RN4CAST / International Hospital Outcomes surveys of nurse staffing and quality (Ball et al., 2014, Sermeus et al., 2011). The two questions were “Were there enough nursing staff to provide quality care on the last shift?” and “Was necessary nursing care left undone (missed) on the last shift because there were too few nursing staff?” In our main analysis, we used the evening responses to these questions, but also performed sensitivity analyses where we instead used afternoon (or evening if missing) reports.

In asking staff to report whether they have enough staff for quality and whether care was missed due to insufficient staff, we are assessing the perceived adequacy of staffing relative to the available staffing. Specifically we ask whether the staffing level was sufficient to produce quality of care and to accomplish all necessary work. In measuring the association between the proportion of long shifts and perceived staffing adequacy, we determine whether this proportion of 12-hour shifts contributes to the perception of adequate staffing. As we adjust for staffing levels in our multivariable models, any association between proportion of 12-hour shifts and adequate staffing indicates that using long shifts is more (or less) likely to produce adequate staffing with any given staffing level. Therefore, the results indicate the relative productivity of the workforce under these varying conditions.

### 2.4 Data cleaning and analysis

We cleaned and processed data and performed statistical analyses in R statistical software V3.5.0 (R Core Team, 2019). The amount of missing data and outliers are recorded in supplementary material table 5. We identified and removed outlying values of staffing shortfall, defined as values outside the ward mean plus or minus three standard deviations (approximately 1.5% of cases). This removed atypical periods when wards were not functioning as normal, e.g. over the Christmas period, and extreme errors in the recorded patients per Safer Nursing Care Tool category. Where there was no record of the number of patients per Safer Nursing Care Tool category for a time period, we used the record from another period on the same day (i.e. substituted morning observations for afternoons and vice versa). We undertook a sensitivity analysis by removing days with missing observations to ensure that this approach did not introduce bias. Since the specialist cancer hospital had mainly long shifts, we performed a sensitivity analysis where we removed this hospital.

For wards that underwent major changes such as moving location, changes to the patient population or bed numbers, we split data and treated them as separate wards. We found some evidence of consistent reverse coding of data inputs (0/1 for yes/no) for “enough staff for quality” in most wards of one hospital. This appeared to result from erroneous staff training. Because it was discovered partway through the study, we developed logical rules to identify wards where this occurred and recoded data. Sensitivity analysis showed that the relationship between staffing adequacy and shortfall was unchanged when these recoded data were excluded and so we retained them to maximise the available sample.

We modelled the relationship between the proportion of long shifts and nurses-in-charge's reports of “enough staff for quality” and “nursing care left undone”. We controlled for registered nurse/assistant staffing shortfall, day of the week, proportion of single rooms, patient turnover, ward specialty (surgical versus medical or mixed) and the number of beds. We fitted multilevel logistic regression models for binary outcomes with random intercepts for hospitals and wards. This accounts for the nested structure of the data (observations within wards, and wards within hospitals). For this we used the glmer (generalised linear mixed effects regression) function from the lme4 package (Bates et al., 2015) in R. We fitted univariable and multivariable models and consequently we investigated whether including the interaction between proportion long shifts and registered nurse/assistant shortfall further improved model fit. We compared the fit of models using the Akaike and Bayesian information criteria, preferring models with lower values, indicating better fit and more parsimonious models (Burnham and Anderson, 2004).

### 2.5 Ethical approval and registration

Ethical approval was granted by the University of Southampton Ethics committee (reference 18809). The study was prospectively registered (ISRCTN 12307968). This study did not require NHS Research Ethics Committee approval because no data were collected directly from patients, and all patient data were pseudoanonymised at source with no sensitive patient data transferred.

## 3 Results

The sample comprised 81 wards, which we converted into 86 pseudo-wards by splitting the data for any wards that underwent major changes (e.g. in size or specialty). Table 1 shows the characteristics of these wards. These were a mixture of medical, surgical and mixed medical/surgical, for example oncology wards. The number of beds per ward ranged from 8 to 63, with 24 on average. Some wards had no single rooms, others had all single rooms, and on average a third of ward beds were in single rooms. Patient turnover (admissions and discharges per worked hour) also varied between wards from 0.01 to 0.34. Some wards had a surplus of staff (according to the Safer Nursing Care Tool) on average across the year, but the mean across wards was a shortfall of 0.4 registered nurse hours per patient day and 0.8 nursing assistant hours per patient day.

**Table 1:** Ward characteristics.

	Mean	Minimum	Maximum
Ward type - medical	39		
- surgical	24		
- mixed	23		
Beds	23.6	8	63
Percentage single rooms	32%	0%	100%
Turnover (mean patients per worked hour)	0.07	0.01	0.34
Registered nurse shortfall (mean hours per patient day)	0.4	-4.5	2.9
Nursing assistant shortfall (mean hours per patient day)	0.8	-1.5	3.4
Mean percentage long shifts	72%	36%	95%
Percentage of evenings reported “Enough staff for quality”	73%	28%	100%
Percentage of evenings reported “Nursing care left undone”	7%	0%	32%

On average across wards, 72% of shifts were long (at least 12 hours), but this ranged between wards from 36% to 95%. There was a similar spread of long shifts for registered nurses as for nursing assistants. The use of long shifts within the same ward varied from day to day, with an average standard deviation of 12% per ward. Since there were no ward days with all short shifts (see Table 2), we used all long shifts as our reference category against which to compare differing degrees of use of long shifts. In our regression models, we used 20% interval widths and combined 0-20% and 20%-40% given the low number of observations in the 0-20% category.

**Table 2:** Distribution of long shifts.

	Ward-days			Wards	
Percentage of long shifts	N	Percentage	Mean percentage of long shifts	N	Percentage
0%	0	0%	0%	0	0%
[0-20%)	88	0.4%	[0-20%)	0	0%
[20-40%)	782	4.0%	[20-40%)	1	1%
[40-60%)	3544	18.0%	[40-60%)	12	14%
[60-80%)	8185	41.7%	[60-80%)	53	62%
[80-100%)	5309	27.0%	[80-100%)	20	23%
100%	1740	8.9%	100%	0	0%

Nurses-in-charge reported having enough staff for quality an average of 73% of the time (ranging across wards from 28% to 100%). Nursing care left undone was reported 7% of the time (range 0-32%). In a cross-classification of long shifts against outcomes, the mean proportion of long shifts per ward is very slightly higher when “enough staff for quality” is reported (74.3% versus 73.7%) and the same is true when no “nursing care left undone” is reported (74.2% versus 72.0%), although this analysis ignores all other variables.

Because these univariable associations do not account for ward/hospital-level effects or other variables that could affect staffing adequacy, we explored these relationships with multi-level logistic regression models, controlling for variables including staffing shortfall, and with ward and hospital as random effects.

For days with a mixture of long and short shifts, the odds of nurses-in-charge reporting enough staff for quality were between 14-17% lower than when all shifts were long (adjusted odds ratios between 0.83-0.86). Although days with mixed shifts were associated with lower odds of reporting that there were enough staff for quality, the relationship did not appear to be linear, with no evidence that odds decreased as the proportion of long shifts decreased. Indeed, the relationship was only statistically significant at the 5% level for proportions of long shifts 60% to 100% although coefficients were similar regardless of the proportion of long shifts below 100% (Table 3, left). The relationship between use of long shifts and reports of nursing care left undone appears to be consistent with this relationship, although the size of effects is less certain since the model did not converge and associations were not statistically significant at the 5% level (Table 3, right). Although statistical significance of the long shift variables altered somewhat in sensitivity analyses, the patterns of results were similar and did not substantively change our conclusions (Supplementary Tables 6-8).

**Table 3:** Outputs of multi-level logistic regression models of the association between the proportion of long shifts and reports of enough staff for quality/nursing care left undone.

	Enough staff for quality				Nursing care left undone (did not converge)			
	Unadjusted odds	Adjusted odds	95% confidence interval	p-value*	Unadjusted odds	Adjusted odds	95% confidence interval	p-value*
100% long shifts (reference)								
80%<= long shifts <100%	0.82	0.83	[0.72, 0.95]	**0.008**	1.09	1.08	[0.81, 1.43]	0.598
60%<= long shifts <80%	0.82	0.85	[0.73, 0.98]	**0.028**	1.08	1.04	[0.78, 1.38]	0.787
40%<= long shifts <60%	0.83	0.86	[0.73, 1.02]	0.086	1.24	1.17	[0.86, 1.59]	0.328
0< long shifts <40%	0.78	0.84	[0.66, 1.07]	0.159	1.44	1.34	[0.90, 2.00]	0.149
Registered nurse shortfall (hours per patient day)	0.95	0.92	[0.90, 0.94]	**0.000**	1.15	1.21	[1.17, 1.26]	**0.000**
Nursing assistant shortfall (hours per patient day)	0.95	0.91	[0.89, 0.94]	**0.000**	1.13	1.20	[1.15, 1.25]	**0.000**
Monday (reference)								
Tuesday	0.92	0.92	[0.81, 1.04]	0.170	0.98	0.98	[0.79, 1.20]	0.818
Wednesday	1.06	1.05	[0.93, 1.19]	0.432	1.05	1.07	[0.87, 1.31]	0.537
Thursday	0.93	0.92	[0.82, 1.05]	0.221	0.99	1.02	[0.83, 1.26]	0.851
Friday	0.98	0.98	[0.86, 1.11]	0.730	1.03	1.04	[0.84, 1.27]	0.745
Saturday	1.08	1.08	[0.95, 1.23]	0.233	0.90	0.90	[0.72, 1.11]	0.329
Sunday	1.32	1.30	[1.15, 1.48]	**0.000**	0.65	0.67	[0.53, 0.84]	**0.000**
Medical or mixed ward (reference)								
Surgical ward	0.84	0.78	[0.45, 1.36]	0.388	1.78	1.99	[1.15, 3.43]	**0.014**
Proportion single rooms	0.55	0.43	[0.13, 1.38]	0.157	1.58	3.08	[0.98, 9.67]	0.054
Turnover (mean patients per worked hour)	0.26	0.78	[0.38, 1.58]	0.485	6.52	1.07	[0.35, 3.28]	0.901
Total beds	1.02	1.02	[0.99, 1.05]	0.326	0.98	0.99	[0.96, 1.02]	0.398
Variance partition coefficient for wards		0.25				0.20		
Variance partition coefficient for hospitals		0.07				0.15		
Akaike Information Criterion		20317				8558		
Bayesian Information Criterion		20466				8708		

⁎ p-values are marked in bold if statistically significant at the 5% level.

**Figure 1:**
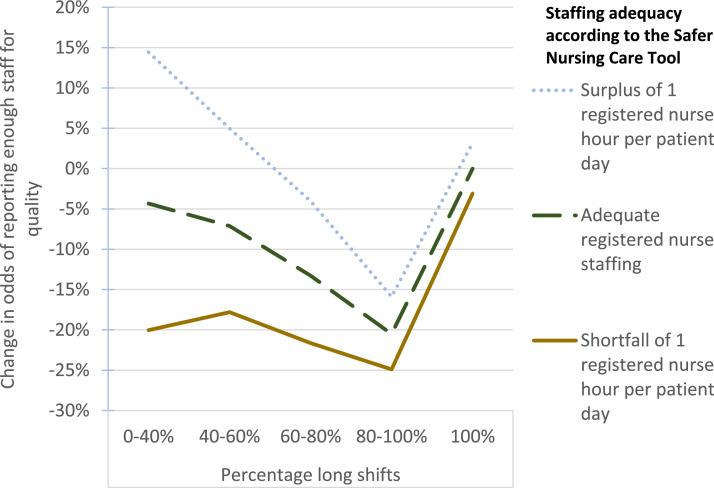
Combined effects of registered nurse staffing shortfall and the proportion long shifts (including interactions) on the odds of reporting enough staff for quality.

We introduced interaction effects between registered nurse/ nursing assistant shortfall and the proportion of long shifts in our models for “enough staff for quality”/ “nursing care left undone” (see Table 4). Although the model fit was worse for nursing care left undone, and not clearly improved for enough staff for quality (Akaike Information Criterion was better, but Bayesian Information Criterion was worse when interactions were included), in both models there was some evidence of interaction effects between staffing shortfalls and the proportion of long shifts. In order to demonstrate the practical significance of these interactions, we plotted the combined effects of registered nurse shortfall and the proportion of long shifts on reports of enough staff for quality in Figure 1.

**Table 4:** Outputs of multi-level logistic regression models of the association between the proportion of long shifts and reports of enough staff for quality/nursing care left undone, including interactions between staffing shortfalls and long shifts.

	Enough staff for quality			Nursing care left undone		
	Adjusted odds	95% confidence interval	p-value*	Adjusted odds	95% confidence interval	p-value*
100% long shifts (reference)						
80%<= long shifts <100%	0.79	[0.69, 0.92]	**0.002**	0.98	[0.73, 1.31]	0.885
60%<= long shifts <80%	0.87	[0.74, 1.01]	0.072	0.91	[0.66, 1.23]	0.528
40%<= long shifts <60%	0.93	[0.77, 1.13]	0.454	0.90	[0.62, 1.29]	0.558
0< long shifts <40%	0.96	[0.69, 1.32]	0.789	1.27	[0.74, 2.18]	0.392
Registered nurse shortfall (hours per patient day)	0.97	[0.93, 1.01]	0.155	1.04	[0.94, 1.14]	0.436
Nursing assistant shortfall (hours per patient day)	0.96	[0.91, 1.02]	0.178	1.04	[0.92, 1.17]	0.513
Monday (reference)						
Tuesday	0.91	[0.80, 1.03]	0.144	0.97	[0.79, 1.20]	0.809
Wednesday	1.04	[0.92, 1.18]	0.499	1.07	[0.87, 1.31]	0.538
Thursday	0.92	[0.81, 1.04]	0.198	1.02	[0.83, 1.25]	0.874
Friday	0.98	[0.86, 1.11]	0.759	1.03	[0.83, 1.27]	0.799
Saturday	1.08	[0.95, 1.23]	0.220	0.89	[0.71, 1.10]	0.273
Sunday	1.31	[1.15, 1.49]	**0.000**	0.66	[0.52, 0.82]	**0.000**
Medical or mixed ward (reference)						
Surgical ward	0.78	[0.45, 1.36]	0.380	1.98	[1.15, 3.43]	**0.014**
Proportion single rooms	0.46	[0.14, 1.48]	0.193	2.65	[0.84, 8.39]	0.098
Turnover (mean patients per worked hour)	0.77	[0.38, 1.56]	0.467	1.13	[0.37, 3.45]	0.825
Total beds	1.02	[0.99, 1.05]	0.319	0.99	[0.96, 1.02]	0.388
**Interaction terms**						
Registered nurse shortfall X 100% long shifts (reference)						
Registered nurse shortfall X 80%<= long shifts <100%	0.98	[0.93, 1.02]	0.309	1.15	[1.03, 1.29]	**0.011**
Registered nurse shortfall X 60%<= long shifts <80%	0.93	[0.89, 0.98]	**0.004**	1.18	[1.06, 1.31]	**0.003**
Registered nurse shortfall X 40%<= long shifts <60%	0.91	[0.86, 0.97]	**0.005**	1.22	[1.08, 1.39]	**0.001**
Registered nurse shortfall X 0< long shifts <40%	0.86	[0.76, 0.98]	**0.019**	1.15	[0.95, 1.40]	0.145
Nursing assistant shortfall X 100% long shifts (reference)						
Nursing assistant shortfall X 80%<= long shifts <100%	0.98	[0.92, 1.04]	0.493	1.14	[1.00, 1.31]	0.058
Nursing assistant shortfall X 60%<= long shifts <80%	0.93	[0.88, 0.99]	**0.028**	1.15	[1.01, 1.31]	**0.035**
Nursing assistant shortfall X 40%<= long shifts <60%	0.92	[0.85, 0.99]	**0.022**	1.20	[1.04, 1.38]	**0.012**
Nursing assistant shortfall X 0< long shifts <40%	0.93	[0.83, 1.05]	0.272	1.12	[0.92, 1.36]	0.267
Variance partition coefficient for wards	0.25			0.20		
Variance partition coefficient for hospitals	0.06			0.15		
Akaike Information Criterion	20313			8560		
Bayesian Information Criterion	20525			8773		

⁎ p-values are marked in bold if statistically significant at the 5% level.

Accounting for interaction effects, on days with a shortfall of one registered nurse hour per patient day, when mixed short and long shifts were used, the odds of reporting enough staff for quality were lower than when there were all long shifts. Likewise, on days with adequate registered nurse staffing, when mixed short and long shifts were used, the odds of reporting enough staff for quality were lower than when there were all long shifts. However, when there was a surplus of one registered nurse hour per patient day and relatively few long shifts (between 0-60%), the odds of reporting enough staff for quality were higher than when there were all long shifts. Regardless of the staffing adequacy according to the Safer Nursing Care Tool, days with between 80-100% long shifts had the lowest odds of reporting enough staff for quality. There is substantial uncertainty in the interaction effect estimates, so these patterns are not conclusive.

## 4 Discussion

Although we found evidence that when there were all long shifts, nurses-in-charge were more likely to report enough staff for quality, the likelihood of reporting adequate staffing did not increase linearly with the proportion of long shifts. Rather, coefficients were similar regardless of the proportion of long shifts below 100%, with odds of reporting enough staff for quality 14-17% lower than when there were all long shifts. The association between long shifts and reports of nursing care left undone were consistent with this finding, but the model did not converge, and confidence intervals were wide. Although including interactions between staffing shortfalls and the proportion of long shifts did not improve model fit, the effect of long shifts did appear to differ according to shortfall, with lower proportions of long shifts associated with benefits when staffing levels were high relative to current norms.

Our finding that nurses-in-charge were less likely to report enough staff for quality on days with a mixture of long and short shifts, than on days with all long shifts, agrees with previous analyses by Griffiths et al. (2019) who found that mixed shift patterns appeared to lead to higher resource use, i.e. it was a less efficient use of staff, than having all short or all long shifts. They found no difference between all short shifts and a high proportion/ all long shifts, but here we could not estimate the effects of all short shifts. One possible explanation for the lower efficiency of mixed shift patterns could be the additional shift overlaps, meaning that more time may be spent handing over information about, rather than caring for, patients Dall'Ora et al., 2019b. When there are mixtures of shift patterns, times for communication between staff are more complicated to organise. There are more changes of staff so more chances for patient information to be lost. Another reason could be that when there are mixed shift lengths, the numbers of staff present on the ward are changing more often over the course of the day, so it may be harder for the nurse-in-charge to keep track of whether there are enough people to do the work.

Evidence for the link between long shifts and nurses-in-charge's reports of nursing care left undone was weaker, with no statistically significant effects at the 5% level. However, the direction of effects was consistent with the “enough staff for quality” model; mixed shift lengths appeared to be associated with higher odds of reporting care left undone. A previous Europe-wide study, the RN4CAST study (Griffiths et al., 2014), which involved surveying 31,627 individual registered nurses, found that working long shifts was associated with more instances of missed care. It may be that the registered nurse has a fuller picture of the care that they left undone, while the nurse-in-charge may only be aware of the most important activities that were missed or postponed.

Although the model fits were not improved, our models including interaction terms suggested the relationship between the proportion of long shifts and staffing adequacy may vary according to the level of staffing. Relative to 100% long shifts, mixed shift patterns are associated with lower perceptions of adequate staffing (in terms of the nurse-in-charge assessing there are enough staff to deliver quality care) unless staffing levels are high relative to current norms. With high staffing, using a lower proportion of long shifts is associated with higher perceptions of staffing adequacy. This is consistent with long shifts having some role in ameliorating the effects of low staffing, but the current norms derived from the Safer Nursing Care Tool are not directly supported by any evidence that such staffing levels are ‘optimal’. Indeed, there is some evidence that benefit accrues to patients from staffing levels higher than these norms (Fagerstrom et al., 2018, Griffiths et al., 2018a) and so higher staffing levels, where a lower proportion of long shifts are associated with a higher chance that staffing is perceived as adequate, could still be preferable (Griffiths et al., 2020b).

According to existing large-scale studies, long shifts are associated with a range of negative consequences for both staff (Dall'Ora et al., 2019a, Thompson, 2019, Dall'Ora et al., 2015) and patients(Ball et al., 2017, Dall'Ora et al., 2019b, Dall'Ora et al., 2019c, Griffiths et al., 2014, Rogers et al., 2004). The original argument for long shifts over short shifts was increasing productivity and reducing costs (Ganong et al., 1976). However, this study adds to the evidence that the mixed shift patterns that have resulted from advocating long shifts are more resource-intensive than using shifts of the same length (Griffiths et al., 2019).

### 4.1 Strengths and Limitations

This was a large study using data from 81 wards in four hospitals in England, so may not generalise to all health systems, although evidence of the link between long shifts and quality of care/missed care exists for other health systems (Griffiths et al., 2014). Our data only allowed us to compare one characteristic of shift work, i.e. long or short, when shift characteristics are more complicated. Other shift characteristics include length of working week, overtime, breaks, rotating shifts and fixed night shifts, which are also associated with staff performance and wellbeing (Dall'Ora et al., 2016). We add to the literature on the effects of nursing shift work by examining the overall ward-level effect of different shift lengths on perceptions of having enough staff to care for patients, rather than considering the effect of individual nurses’ shifts. However, this judgement is subjective and may not directly reflect the experiences of individual nurses delivering care, or indeed the patients. The nurse-in-charge has a global view of the shift while individual nurses have a more granular view. Therefore, individual nurses may notice the first signs of not having enough staff for quality, and notice every aspect of care they left undone, while the nurse-in-charge may only be made aware of the more major incidents and critical instances of missed care. Moreover, we asked nurses-in-charge to only report missed care/poor quality if it related to having insufficient staff. This means that finding relationships between long shifts and staffing adequacy as reported by the nurse-in-charge adds further weight to existing evidence, since the relationship is so strong that it is noticeable at a ward level. This could also explain the lack of statistical significance in some cases. The measurement of productivity in nursing is problematic and our study has only done so using a subjective assessment. While missed care can be considered a direct measure of productivity, quality of care cannot, although it is important in understanding quality-adjusted productivity.

### 4.2 Conclusions

Rather than a clear distinction between wards using short and long shifts, we found that a mixed pattern operated on most days and wards, with no wards using all short shifts. We found that when wards use exclusively long shifts rather than a mixture, nurses-in-charge are more likely to judge that they have enough staff. Consistently with previous evidence, we found that using mixed shift lengths appears to be more resource-intensive than using shifts all the same length. However, the adverse effects of mixed shifts on perceptions of staffing adequacy may be reduced or eliminated by higher staffing levels.

Future research should include a qualitative exploration of nurses-in-charge's perceptions of 12-hour shifts and staffing adequacy and missed care, which is largely missing in the literature as the few qualitative studies on the topic are only with registered nurses and nursing assistants but not with managers. Also it would be important to further understand the underlying mechanisms which lead mixed shift patterns to be associated with lower staffing adequacy.

## CRediT authorship contribution statement

**Christina Saville:** Methodology, Formal analysis, Validation, Writing - original draft. **Chiara Dall'Ora:** Conceptualization, Methodology, Writing - review & editing, Supervision. **Peter Griffiths:** Conceptualization, Methodology, Writing - review & editing, Supervision, Funding acquisition.

## Appendix Supplementary materials

Image, application 1
