# Supplementary material for: The association between 12-hour shifts and nurses-in-charge's perceptions of missed care and staffing adequacy: a retrospective cross-sectional observational study
Source: Int J Nurs Stud. 2020 Dec;112:103721. doi: 10.1016/j.ijnurstu.2020.103721 (PMC7695680; doi:10.1016/j.ijnurstu.2020.103721)
Supplement: Supplementary file 1 [file mmc1.docx]

Table 5: Data sample sizes

|  | Possible ward days | Valid answer to "enough staff for quality" | | Valid answer to "nursing care left undone" | |  | Possible ward mornings and afternoons | Safer Nursing Care Tool rating recorded | | SNCT rating recorded, after substituting end of period if beginning missing | | SNCT ratings, with outliers removed | |  | Shortfall recorded for both morning and afternoon | | Shortfall recorded for both morning and afternoon after substituting other period if missing | |  |  | Linked data (shortfall and "enough staff for quality") | | Linked data (shortfall and "nursing care left undone") | |
| --- | --- | --- | --- | --- | --- | --- | --- | --- | --- | --- | --- | --- | --- | --- | --- | --- | --- | --- | --- | --- | --- | --- | --- | --- |
|  | ward-days | ward-days | % | ward-days | % |  | ward-periods | ward-periods | % | ward-periods | % | ward-periods | % |  | ward-days | % | ward-days | % |  |  | ward-days | % | ward-days | % |
| A | 6419 | 5740 | 89% | 5498 | 86% |  | 12838 | 6958 | 54% | 12266 | 96% | 12031 | 98% |  | 5703 | 89% | 6328 | 99% |  |  | 5699 | 89% | 5460 | 85% |
| B | 10130 | 8140 | 80% | 8140 | 80% |  | 20260 | 9182 | 45% | 17008 | 84% | 16746 | 98% |  | 7040 | 69% | 9706 | 96% |  |  | 7943 | 78% | 7943 | 78% |
| C | 4113 | 2677 | 65% | 2684 | 65% |  | 8226 | 6769 | 82% | 7198 | 88% | 7097 | 99% |  | 3313 | 81% | 3784 | 92% |  |  | 2613 | 64% | 2620 | 64% |
| D | 6897 | 3417 | 50% | 3404 | 49% |  | 13794 | 11237 | 81% | 11879 | 86% | 11726 | 99% |  | 5428 | 79% | 6298 | 91% |  |  | 3393 | 49% | 3380 | 49% |
| All | 27559 | 19974 | 72% | 19726 | 72% |  | 55118 | 34146 | 62% | 48351 | 88% | 47600 | 98% |  | 21484 | 78% | 26116 | 95% |  |  | 19648 | 71% | 19403 | 70% |

*Table 6: Outputs of multi-level logistic regression models of the association between the proportion of long shifts and reports of enough staff for quality/nursing care left undone* ***without specialist cancer hospital***

|  | Enough staff for quality | | |  | Nursing care left undone | | |
| --- | --- | --- | --- | --- | --- | --- | --- |
|  | Adjusted odds | 95% confidence interval | p-value |  | Adjusted odds | 95% confidence interval | p-value |
| 100% long shifts (reference) |  |  |  |  |  |  |  |
| 80%<= long shifts <100% | 0.81 | [0.67, 0.98] | 0.030 |  | 1.25 | [0.90, 1.74] | 0.178 |
| 60%<= long shifts <80% | 0.81 | [0.67, 0.98] | 0.029 |  | 1.24 | [0.89, 1.73] | 0.201 |
| 40%<= long shifts <60% | 0.83 | [0.67, 1.02] | 0.070 |  | 1.38 | [0.97, 1.96] | 0.072 |
| 0< long shifts <40% | 0.81 | [0.62, 1.06] | 0.122 |  | 1.59 | [1.03, 2.44] | 0.036 |
| Registered nurse shortfall (hours per patient day) | 0.89 | [0.86, 0.91] | 0.000 |  | 1.23 | [1.18, 1.28] | 0.000 |
| Nursing assistant shortfall (hours per patient day) | 0.89 | [0.86, 0.91] | 0.000 |  | 1.21 | [1.16, 1.26] | 0.000 |
| Monday (reference) |  |  |  |  |  |  |  |
| Tuesday | 0.88 | [0.77, 1.01] | 0.072 |  | 0.96 | [0.77, 1.18] | 0.680 |
| Wednesday | 1.01 | [0.88, 1.16] | 0.870 |  | 1.07 | [0.86, 1.32] | 0.547 |
| Thursday | 0.92 | [0.80, 1.06] | 0.244 |  | 1.05 | [0.85, 1.30] | 0.642 |
| Friday | 0.97 | [0.85, 1.11] | 0.679 |  | 1.01 | [0.82, 1.25] | 0.905 |
| Saturday | 1.02 | [0.89, 1.18] | 0.756 |  | 0.92 | [0.74, 1.14] | 0.448 |
| Sunday | 1.32 | [1.14, 1.52] | 0.000 |  | 0.64 | [0.51, 0.81] | 0.000 |
| Medical or mixed ward (reference) |  |  |  |  |  |  |  |
| Surgical ward | 0.77 | [0.42, 1.40] | 0.394 |  | 2.06 | [1.16, 3.65] | 0.014 |
| Proportion single rooms | 0.61 | [0.10, 3.74] | 0.597 |  | 4.13 | [0.78, 21.89] | 0.095 |
| Turnover (mean patients per worked hour) | 0.96 | [0.45, 2.05] | 0.915 |  | 1.08 | [0.35, 3.36] | 0.898 |
| Total beds | 1.02 | [0.98, 1.05] | 0.378 |  | 0.98 | [0.95, 1.02] | 0.343 |

*Table 7:* *Outputs of multi-level logistic regression models of the association between the proportion of long shifts and reports of enough staff for quality/nursing care left undone using* ***afternoon (or evening if missing) responses***

|  | Enough staff for quality | | |  | Nursing care left undone | | |
| --- | --- | --- | --- | --- | --- | --- | --- |
|  | Adjusted odds | 95% confidence interval | p-value |  | Adjusted odds | 95% confidence interval | p-value |
| 100% long shifts (reference) |  |  |  |  |  |  |  |
| 80%<= long shifts <100% | 0.88 | [0.70, 1.11] | 0.289 |  | 1.32 | [0.83, 2.10] | 0.241 |
| 60%<= long shifts <80% | 0.90 | [0.77, 1.06] | 0.206 |  | 1.26 | [0.92, 1.74] | 0.154 |
| 40%<= long shifts <60% | 0.93 | [0.81, 1.07] | 0.311 |  | 1.20 | [0.90, 1.60] | 0.205 |
| 0< long shifts <40% | 0.89 | [0.78, 1.02] | 0.097 |  | 1.17 | [0.89, 1.54] | 0.269 |
| Registered nurse shortfall (hours per patient day) | 0.91 | [0.89, 0.92] | 0.000 |  | 1.17 | [1.12, 1.22] | 0.000 |
| Nursing assistant shortfall (hours per patient day) | 0.89 | [0.87, 0.92] | 0.000 |  | 1.18 | [1.13, 1.24] | 0.000 |
| Monday (reference) |  |  |  |  |  |  |  |
| Tuesday | 0.99 | [0.88, 1.12] | 0.872 |  | 1.04 | [0.84, 1.30] | 0.699 |
| Wednesday | 1.10 | [0.97, 1.24] | 0.134 |  | 0.92 | [0.73, 1.15] | 0.451 |
| Thursday | 1.02 | [0.91, 1.16] | 0.697 |  | 0.93 | [0.74, 1.16] | 0.523 |
| Friday | 0.98 | [0.86, 1.10] | 0.689 |  | 1.02 | [0.82, 1.28] | 0.860 |
| Saturday | 1.05 | [0.93, 1.19] | 0.410 |  | 1.08 | [0.87, 1.35] | 0.489 |
| Sunday | 1.39 | [1.23, 1.58] | 0.000 |  | 0.79 | [0.62, 1.00] | 0.050 |
| Medical or mixed ward (reference) |  |  |  |  |  |  |  |
| Surgical ward | 0.71 | [0.42, 1.18] | 0.185 |  | 2.03 | [1.16, 3.58] | 0.014 |
| Proportion single rooms | 0.47 | [0.16, 1.39] | 0.173 |  | 2.02 | [0.63, 6.48] | 0.239 |
| Turnover (mean patients per worked hour) | 0.80 | [0.40, 1.59] | 0.521 |  | 0.77 | [0.25, 2.41] | 0.651 |
| Total beds | 1.01 | [0.99, 1.04] | 0.357 |  | 1.00 | [0.97, 1.04] | 0.801 |

*Table 8: Outputs of multi-level logistic regression models of the association between the proportion of long shifts and reports of enough staff for quality/nursing care left undone* ***(Days with missing observations removed)***

|  | Enough staff for quality | | |  | Nursing care left undone (did not converge) | | |
| --- | --- | --- | --- | --- | --- | --- | --- |
|  | Adjusted odds | 95% confidence interval | p-value |  | Adjusted odds | 95% confidence interval | p-value |
| 100% long shifts (reference) |  |  |  |  |  |  |  |
| 80%<= long shifts <100% | 0.87 | [0.68, 1.12] | 0.285 |  | 1.40 | [0.93, 2.13] | 0.110 |
| 60%<= long shifts <80% | 0.86 | [0.72, 1.02] | 0.088 |  | 1.17 | [0.85, 1.63] | 0.338 |
| 40%<= long shifts <60% | 0.84 | [0.72, 0.98] | 0.024 |  | 1.08 | [0.80, 1.46] | 0.618 |
| 0< long shifts <40% | 0.83 | [0.71, 0.96] | 0.012 |  | 1.08 | [0.80, 1.45] | 0.614 |
| Registered nurse shortfall (hours per patient day) | 0.92 | [0.90, 0.94] | 0.000 |  | 1.19 | [1.14, 1.24] | 0.000 |
| Nursing assistant shortfall (hours per patient day) | 0.92 | [0.90, 0.94] | 0.000 |  | 1.19 | [1.14, 1.24] | 0.000 |
| Monday (reference) |  |  |  |  |  |  |  |
| Tuesday | 0.90 | [0.79, 1.03] | 0.128 |  | 0.98 | [0.79, 1.22] | 0.869 |
| Wednesday | 1.02 | [0.89, 1.16] | 0.772 |  | 1.07 | [0.87, 1.33] | 0.507 |
| Thursday | 0.90 | [0.79, 1.02] | 0.101 |  | 1.02 | [0.82, 1.26] | 0.859 |
| Friday | 0.96 | [0.84, 1.09] | 0.521 |  | 1.04 | [0.83, 1.29] | 0.738 |
| Saturday | 1.09 | [0.95, 1.24] | 0.234 |  | 0.92 | [0.73, 1.15] | 0.444 |
| Sunday | 1.26 | [1.10, 1.45] | 0.001 |  | 0.68 | [0.54, 0.87] | 0.002 |
| Medical or mixed ward (reference) |  |  |  |  |  |  |  |
| Surgical ward | 0.78 | [0.44, 1.38] | 0.388 |  | 1.96 | [1.11, 3.48] | 0.021 |
| Proportion single rooms | 0.41 | [0.12, 1.38] | 0.152 |  | 2.89 | [0.87, 9.58] | 0.082 |
| Turnover (mean patients per worked hour) | 0.91 | [0.41, 2.05] | 0.829 |  | 1.16 | [0.33, 4.12] | 0.813 |
| Total beds | 1.03 | [0.99, 1.06] | 0.126 |  | 0.98 | [0.95, 1.01] | 0.236 |
